# Barriers to surveillance and control of re-emergence of the Chagas disease vector *Triatoma infestans* in Arequipa, Peru

**DOI:** 10.1371/journal.pntd.0013373

**Published:** 2025-08-07

**Authors:** Laura D. Tamayo, Valerie A. Paz-Soldán, Carlos E. Condori Pino, Fernando S. Malaga Chavez, Michael Z. Levy, Raquel Gonçalves

**Affiliations:** 1 Zoonotic Disease Research Laboratory, One Health Unit, School of Public Health and Administration, Universidad Peruana Cayetano Heredia, Arequipa, Perú; 2 Department of Tropical Medicine and Infectious Disease, School of Public Health and Tropical Medicine, Tulane University, New Orleans, Louisiana, United States of America; 3 Executive Directorate of Environmental Health, Gerencia Regional de Salud, Arequipa, Perú; 4 Department of Biostatistics, Epidemiology and Informatics, University of Pennsylvania, Philadelphia, Pennsylvania, United States of America; 5 Department of Biology, University of Pennsylvania, Philadelphia, Pennsylvania, United States of America; University of Florida College of Medicine, UNITED STATES OF AMERICA

## Abstract

Vector control is usually designed as a top-down system with limited capacity to respond to the unique characteristics of each epidemiological setting, or to adjust to routine stressors that challenge ongoing programs, such as resource limitations and competing priorities. Here, we investigated barriers in Chagas disease vector surveillance and control systems in Arequipa, Peru. We conducted in-depth interviews and a focus group with key stakeholders (n = 32) at different levels of the health system and community. For interviews, we used process maps to illustrate the workflow for passive and active surveillance. Additionally, we held a focus group with vector control specialists to present the findings from the interviews and discuss the results. We identified barriers at each step of the process, including systemic, operational, financial, and policy limitations. For passive surveillance, community participation was limited by practical challenges in capturing the insect and uncertainty about the pathways to report it. Systemic barriers were related to the use of a data system that did not meet the needs for recording and managing data on vector control activities. At the policy level, the establishment of quotas on the number of houses staff are required to inspect ignores important determinants for infestation and lacks an appropriate sampling design. We discuss the impact of the reported barriers to effective conduction of surveillance and control activities and the initiatives and strategies that have been designed and assessed to bridge these gaps in order to collaboratively design a more resilient health system.

## Author summary

Vector control strategies are usually based on hierarchical systems, resulting in the limited capacity to adapt to particular epidemiological scenarios or adjust to routine stressors. In Arequipa, Peru, despite successful control of the insects that transmit Chagas disease, challenges to sustain vector surveillance and control activities are numerous. We examined the barriers to sustained vector control by conducting in-depth interviews and focus groups with multiple stakeholders at different levels of the system. We identified significant barriers at each step of the passive and active surveillance processes, resulting in limited potential to respond to infestations and loss of information. At the individual level, barriers to reporting infestation included difficulties in capturing the vector and uncertainty about where or to whom to report it. At the operational level, we identified barriers regarding the quality and consistency of house inspections, as well as competing priorities, such as multiple tasks assigned to vector control specialists. At the system level, the use of an inadequate Health Information System (HIS) with no structure to record relevant information related to vector control activities. At the policy level, establishment of quotas on the number of houses to inspect without considering appropriate sampling. Development of resilient health systems is a collective endeavor involving affected communities, health staff and actors from multiple sectors. It is urgent to review strategies to better design resilient control systems for vector-borne diseases.

## Introduction

Vector control programs are critical for the prevention and control of many vector-borne diseases. Despite recognition of barriers to existing vector control programs among those managing them on the ground, research on the implementation and operational challenges of these programs remains limited [1]. Time and space for reflection within the system—necessary to identify barriers and develop solutions—are often missing, due to a lack of data, motivation, or an entrenched system that favors traditional strategies over change [2].

Between 2003 and 2018, a vector control campaign – consisting of an initial preliminary planning phase, an attack phase involving two rounds of indoor residual insecticide application in nearly all houses (regardless of infestation status) to eliminate *Triatoma infestans* from the affected areas, and post-spray surveillance– was conducted in the city of Arequipa, Peru, to eliminate *Triatoma infestan*s, the only local insect vector of Chagas disease [3]. Following the campaign, a community-based surveillance system was gradually established, employing both passive and active surveillance methods. In the passive approach, residents are required to capture insects and deliver them to designated health facilities for identification. Once *T. infestans* is confirmed, a vector control specialist inspects the infested house and neighboring homes, subsequently spraying as needed. If *T. infestans* are detected in additional houses, adjacent houses are inspected until the vector is no longer detected [4].

In active surveillance, Vector Control Specialists (VCS) are assigned a quota (20% of households under their supervision) to inspect. Outside of research projects [3,5,6], there is no guidance on *which* households to visit, leaving the selection to the VCS’s discretion. Monthly reports on home inspections are submitted to the Arequipa-Caylloma health network manager.

Our study aimed to examine and depict the barriers within the complex system of triatomine surveillance and control in Arequipa, Peru. We conducted semi-structured interviews with a diverse range of system stakeholders, as well as a focus group with vector control specialists, to gather additional insights and perspectives [7]. By triangulating these sources, we moved beyond isolated assessments to co-design more effective and sustainable solutions, enabling the development of more informed and impactful strategies for triatomine surveillance and control in Arequipa.

## Methods

### Ethics statement

The research protocol received approval from the ethical review committees at Universidad Peruana Cayetano Heredia (Approval number: 103096) and the University of Pennsylvania (Approval identification number: 833122). Before participation, all individuals provided written consent to take part in the study.

### Study setting

The study was conducted in the Peruvian city of Arequipa (population ~1 million), located in the Andes mountains, south of the capital (Lima). The healthcare system is overseen by two distinct entities: the “Gerencia Regional de Salud” (GERESA), the regulatory body equivalent to a Regional Health Department, and the “Red de Salud Arequipa-Caylloma” (Arequipa-Caylloma health network), the large health network which manages smaller health networks, or “microredes”, to streamline resource allocation and administration within the city. The microredes are clusters of primary care health facilities grouped by geographic proximity, responsible for serving a defined population residing in relative proximity to these facilities.

Most health facilities have a “Vector Control Specialist” (VCS) overseeing vector surveillance and control activities in their health network, reporting to both the facility manager and to the GERESA vector control program authority. VCSs are generally technicians who were trained during the Chagas disease control campaign to identify triatomine vectors and perform insecticide spraying. After the campaign, many were hired under permanent contracts by the Ministry of Health and received further training in environmental health and sanitary inspection. VCSs are typically assigned to the same jurisdiction in which they were originally deployed. A few facilities lack a VCS; in these cases, a designated “vector point person” relays vector-related reports to a nearby VCS or directly to the vector control program authority in the Arequipa-Caylloma health network.

Vector-borne *T. cruzi* transmission in the city of Arequipa, although not yet officially declared disrupted, has been at an undetectable level for some years. The highest prevalence in the city (4.7% in school children tested in 2005) [8] and around 5% in the population [9] occurred in the south western districts of Tiabaya, Sachaca and Hunter which were treated by 2006, which is also the last year a case of acute Chagas disease was reported [10]. From 2006-2009 the campaign treated districts in which 25.6% of treated households were infested with triatomines, but *T. cruzi* was found in vectors of only 80 (4.1%) of these infested houses, and prevalence of human infection in these households with *T. cruzi* was only 2.6% [11]. The campaign was paused from 2009 to 2011. In 2012 a district in the northeast of the city, Mariano Melgar, was treated. The presence of *T. cruzi* in vectors occurred in very small ‘micro-epidemics’ and the prevalence of infection in the population was 1.4%, many of the infections were not associated with the ‘micro-epidemics’ and likely occurred elsewhere [12].

Post-vector control campaign surveillance efforts primarily focused on passive surveillance, which successfully detected and eliminated most of the residual infestation foci. Following the campaign, a series of data-driven surveillance schemes were piloted in multiple districts of the city, involving the search for the insect in over 8,000 households [3,5,6]. These efforts resulted in the detection of only three infested households. More recently, a study testing a new paradigm for vector surveillance, inspired by the mammalian immune system, detected 28 infested households out of 136,273 houses under surveillance [13]. Again, no vectors were found to be carrying *T. cruzi*.

### Sampling, recruitment and data collection

We investigated the perceptions of different stakeholders regarding barriers in triatomine vector surveillance and control in Arequipa through in-depth interviews and focus group. We used purposive sampling to obtain a range of perspectives regarding different components of the passive and active surveillance system. Interviews were conducted until saturation was reached. We recruited key stakeholders at various levels of the system, including community members, vector control specialists, regional health authorities, and two authorities who had been or, were at the time of the study, were part of the vector control unit at the Regional Health Department. Recruited personnel from the Ministry of Health and some community health workers had been working with the research team for many years, but there was no prior relationship with recruited community members. All recruited stakeholders agreed in participate.

**Fig 1:**
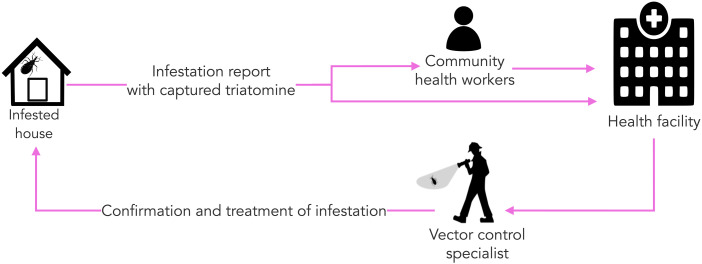
Process map of the passive Chagas disease vector surveillance system in Arequipa, Peru, used to guide participants during the interviews.

Our objective was explicitly communicated repeatedly before and during the interviews: to establish a collective understanding of systemic barriers for triatomine vector surveillance and control. Participants were invited to share their experiences; no personal details about the interviewee were discussed. Interviews were conducted in a private space in workplaces and, in the case of community health workers and community members, in their homes to ensure privacy. We (LDT, VPS, RG, CCP) conducted in-depth interviews using process maps — visual representations of workflows, responsibilities, and decision points — to illustrate how passive and active surveillance systems operate. These visual aids, commonly used in healthcare to analyze and improve complex systems [14], facilitated discussions with interviewees. By referring to each step in the surveillance process, participants were encouraged to identify and elaborate on the specific challenges they encounter. Fig 1 shows the passive surveillance flowchart used during the interviews.

Some interviewees, especially community members, community health workers, and social workers (who oversee the community health workers), had limited awareness of the surveillance system processes. We, therefore, developed a structured interview guide tailored to these participants, focusing on their experiences and challenges within the surveillance system (S1 File). Interviews were conducted until thematic saturation was reached — the point at which no new insights emerged and additional data collection became redundant [15].

In addition, we conducted a focus group (LDT, VPS) with vector control specialists to present the findings from the interviews and discuss the results. During the focus group, we showed the flow charts with the barriers identified in each section of the surveillance process. The specialists were asked whether they had already considered these barriers and if they encountered any additional ones not previously described. This focus group provided insights from professionals directly involved in vector control activities and helped refine our understanding of the practical challenges of improving the system. Semi-structured interviews and focus groups were conducted between November 2021 and March 2024.

### Data management and analysis

We audiotaped and transcribed all interviews and the focus group. Only one respondent requested not to be audiotaped, so we took detailed notes instead. As the interviews progressed, either the interviewer or the respondent filled out blank process maps used to guide discussions on barriers in passive and active surveillance. Based on an initial reading of transcripts, we developed a codebook using both deductive codes (steps of the process maps) and inductive codes (themes emerging from participant responses). This iterative process resulted in a final code tree, which organized the code into categories and subcategories and was later illustrated through flow charts to support interpretation [16]. A team of four individuals double-coded each transcript, refining the codebook iteratively to ensure consistency and accuracy in identifying key themes. Interviews and focus group transcripts were managed, coded, and analyzed using Dedoose (version 9.0.90) [17].

## Results

A total of 32 individuals participated in the interviews and focus groups (see Table 1). None of the individuals invited to participate refused to take part in the study.

**Table 1:** Number of participants recruited and target recruitment by participant role.

Role of participant	Total Recruited	Targeted Recruitment
Vector control specialist	11	At least one VCS in each “microred” (small health network)
Health authorities	4	All surveillance system authorities (and who also manage VCSs)
Social workers and Community health workers	5	Recruit until saturation related to the Chagas surveillance system reached
Community members who reported a home infestation	10	All community members who had reported triatomine infestations in their homes through Health facilities or AlertaChirimacha between November 2021 and March 2024
Community members who did not report a home infestation	2	Community members whose homes were found to be infested through routine inspections surrounding infested homes, but who had not themselves reported the infestation
**Total**	**32**	

**Fig 2:**
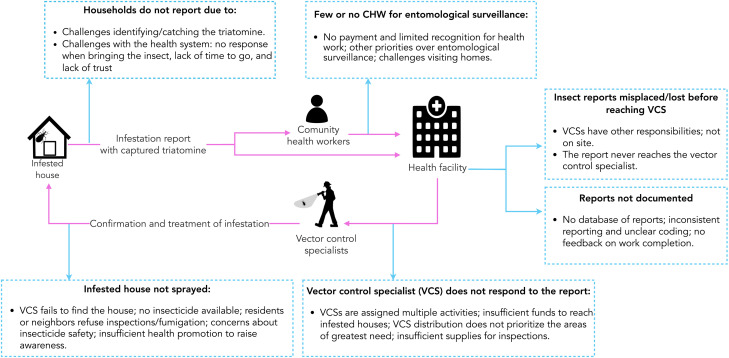
Flowchart of challenges in passive surveillance for triatomine vectors in Arequipa, Peru.

**Fig 3:**
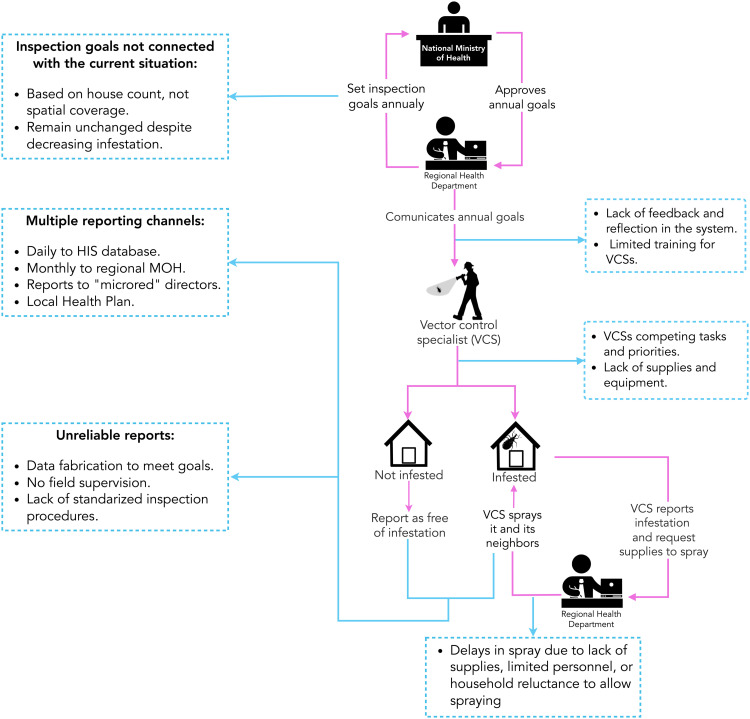
Flowchart of active surveillance for Chagas disease vectors in Arequipa, Peru. Identified barriers are highlighted in light blue boxes.

We identified multiple barriers at each step of the passive and active surveillance processes, resulting in delays or inadequate response to reports, loss of information, and ultimately, a surveillance system that is not working efficiently. To avoid repetition, we present detailed issues for each step of the passive and active systems in the two figures (Figs 2 and 3), while the text focuses on the main themes common to both surveillance systems: 1) systemic challenges and operational barriers, and 2) resource allocation and policy limitations. Additionally, we highlight – and start with – one theme specific to the passive surveillance system: community engagement and reporting challenges.

### Community engagement and reporting challenges

#### Reporting an infestation directly to the health facility.

Interviews revealed four main barriers to reporting triatomines to the local VCSs: 1) lack of awareness among community members about identifying the insect and its potential danger; 2) difficulties in capturing it; 3) uncertainty about where or to whom to report; and, 4) receiving conflicting information at health facilities. Currently, community members are expected to capture the insect (usually in a plastic bag or bottle) and bring it to a health facility. The most commonly reported challenge was difficulty in recognizing or capturing the triatomine:

*“...I used to see it [*triatomine bug*], but I never thought it was a Chirimacha [*common name for triatomines*], nor did I know that this insect was bad.” (Community member)*

*“But they told me, ‘bring it [the Triatomine bug] to me in a bottle’. But that day, as I said, I [thought I] killed it. So I left it. And by the next day, it wasn’t there, it hadn’t died and had gone.” (Community member)*

Participants also reported a lack of knowledge about how and where to report infestations. They had difficulties finding time to visit the health center, and prior lack of response from the health system diminished their motivation to do so. One community member who experienced an infestation but did not report it mentioned mistrust in government institutions:

*“Yes, yes. We knew we had to go to report it, but sometimes, always when we go, in any government institution… there is always a delay… and there is a lot of paperwork, a lot of procedures… so you prefer to pay for your own fumigation.*” *(Community member)*

#### Reporting an infestation through Community Health Workers (CHWs).

Both CHWs, who can report household infestations, and social workers, who manage the CHWs, noted significant barriers to their work, particularly the absence of financial support and recognition from the health system. This lack of support hinders CHWs’ effectiveness and motivation. As one social worker explained:

“*[CHWs] do not have money for public transportation, they do not have credentials, their [work] vest is too small and people do not have material, hats, or backpacks…”*

Most community members did not know their CHW or their role. Additionally, most VCSs and those overseeing the vector-borne strategy indicated that CHWs often prioritize incentive-based activities, like a national anemia program, which provides CHWs with a stipend, over vector surveillance. Many stakeholders, including social workers and VCSs, mentioned that CHWs are often not well-known within the health system, partly due to high staff turnover within the system, and are not fully integrated into the vector surveillance system. One VCS described:

*“If you ask me to bring you a [CHW], I don’t even know their address… I only know them when they come to a health promotion meeting, and later I might see them again when it’s Chagas week in November when they tell us we have to come to the regional government with community health workers. That day, I go to the [*the social worker*], and say, ‘please, assign me some [CHWs], and I’ll bring them’”. (Vector control specialist)*

### Systemic challenges and operational barriers

#### Delays and mismanagement in reporting.

Community members reported significant delays in responses to infestation reports, often requiring multiple visits to the health facility before action was initiated. When VCSs were absent, other health staff mishandled reports by misidentifying the triatomine, dismissing the report, or failing to relay the information to the VCS. In some cases, residents were asked to return later or the next day to submit the insect.

*“... if I am not there, sometimes they send it [a triatomine] to the laboratory, other times it stays in the admission area and sometimes it gets lost... perhaps I am told they brought one, but I don’t remember because the next day they cleaned, saw a bag with an insect and maybe threw it away.” (Vector control specialist)*

***Interviewer*** *: When the community member arrives with a chirimacha, and it is received in the admission area, do they take it to you or does it go to the laboratory? Is there a sheet or documentation of everything that has happened [hand off of the triatomine]?*

***Vector control specialist*** *: No..., no, there is no record, we should do better about that.*

#### Resource constraints.

Vector control specialists are often tasked with other activities, such as patient admission, logistics, triage, and water and sanitation quality control, as well as responding to other zoonotic disease reports. VCSs reported finding it difficult to refuse these tasks since they are assigned by their direct supervisor. They also lack resources, including equipment, supplies, and transportation funds, to carry out inspections or spraying.

#### Resident reluctance and integrity concerns.

In addition to challenges reaching the field for surveillance, VCSs encounter barriers during inspections, such as residents’ fear and reluctance to let them in their homes, due to growing insecurity. This occurs despite prior coordination with the health promotion unit, which works to raise community awareness about the importance of surveillance and allowing VCSs’ entry. One VCS described:

*“Yes, insecurity, insecurity is strong... that is one of the biggest obstacles, right now [...]. That is the biggest problem, they are reluctant, right? They are afraid. People live now with a lot of fear, a lot of fear. They don’t open the door easily or if they answer, they answer from the roof or the window.” (Vector control specialist)*

Neighbors of an infested index house sometimes refuse inspections and treatment, complicating efforts to control infestation foci. One VCS also observed reluctance to allow spraying due to previous negative experiences:

“*Another thing that sometimes happens is that they [community members] are afraid, they are not very confident [of the insecticide]... [We are asked:] ‘What are we going to fumigate and with what?’ Why? ‘Because the previous neighbor or the previous fumigations had... what is it called? Hypersensitivity to insecticide…’ and no, they don’t want [to fumigate]*.” *(Vector control specialist)*

Both VCSs and health authorities raised concerns about the quality and consistency of house inspections – and even if they are being conducted at all. They noted some VCSs no longer perform thorough household inspections. Some only ask residents about triatomines in their home, others enter only parts of the home, whereas others might perform very quick, superficial checks. Authorities also reported instances in which some VCSs falsified data to meet established goals:

“*[One vector control specialist] would come with his little sheet of paper, because we have to have our tangible proof [of inspected houses]. At some point, they told us that homeowners had to sign a paper. But, how do I believe him? Because I have found several papers from previous vector control specialists, I don’t know how they did it, but the code [house identifier] did not match.. I went there myself once because as I am responsible: [the codes] did not match. They didn’t match at all. That’s my problem. So I told [the VCS]: ‘If you don’t do it right, I will always check your little sheet of paper [documentation of inspected house] I’ll do random checks’. And some may respond, ‘Yes, yes you are right, excuse me’. [I tell them]: ‘Don’t fool me, because I know my area’. So I tell you, maybe they made up data to cover the quotas that they had?... I don’t know.*” (Lead vector control specialist)

#### Competing priorities and quality related to the work of vector control specialists.

VCSs report to both the health facility manager and the vector control authority, juggling competing tasks such as sanitation, water quality control, health facility assistance, and vector surveillance. As a result, VCSs feel pressured to complete tasks quickly, often with minimal effort, to meet their goals:

*“Many times the work is based on the convenience of the health staff and I would imagine that the inspection, if they do it at all, is going to be as quick as possible...” (Health Authority)*

The frequent turnover of facility managers aggravates this problem by assigning non-surveillance tasks to the VCSs. A health authority noted that each new manager has different priorities for VCSs, impacting their ability to focus on active surveillance. As a result, the health authority often has to “fight” to ensure VCSs fulfill their surveillance duties:

*Interviewer: And the change of [health facility] managers, how much does it affect surveillance?*

*Health authority: “It affects because a new manager comes in with a new idea. ‘Ah, right! You, for example, you are not going to do that, you are going to do other things, because I need here in this service because here I have no one to do it, so you are going to do it.’ ‘But, doctor, I have my [surveillance] activities’. ‘Just leave those activities!’ There is no importance given to the [surveillance] program on behalf of the managers.”*

### Resource allocation and policy limitations

#### Budget allocation determination at the national and regional levels.

The Ministry of Economy and Finance distributes the budget for zoonotic diseases across government levels. Approximately 22% remains with the National Health institutions (MINSA, CDC, INS) 74.8% is allocated to regional governments, and 3.2% to local governments (data from 2022) [18]. To secure funding, the Regional Health Department (GERESA) must plan and budget for active surveillance activities in line with the technical standards set by the National Ministry of Health. However, regional health authorities reported that the allocated budget is usually less than requested, forcing them to re-adjust their plans. The Peruvian health system incorporates ‘pay for performance’, in which budget allocation is based on the completion of established activities or specified goals, but the budget is often reduced even if goals are purportedly met. One health authority describes:

*Interviewer: So what they give you depends on your results?*

*Health authority: No, no. What they call pay for performance… I think that was the original idea: if you give me health results, then I increase your budget, as a ‘reward’ so that you can continue to carry out your activities and move forward. But no, you meet the goals, and they don’t give you anything, they reduce it [the budget]. Then they say: ‘but you are not complying.’ Of course, I am not complying because I have no means to comply. ‘No, but you must comply in order to…’ How am I going to comply?! If I have no money to be able to comply, how do you want me to comply? [You want me to] make it up just to look good?*

On the other hand, regional governments allocate their funds based on their priorities. While part of the budget is reserved for nationally prioritized health strategies, currently anemia and tuberculosis, the remaining funds are distributed at the discretion of regional governments. Local health authorities for vector-borne and zoonotic diseases noted that Chagas and other zoonotic diseases are often not prioritized, leading to competition between health units to maintain or increase their budgets. One local health authority elaborated further:

*“It would be different if, say, we had a conversation, raised awareness, or did some advocacy with the regional government so that they could increase our budget. We don’t want billions, but we do want to have enough to sustain the existing achievements. For Chagas, we have great achievements, but these can’t be sustained because there is not enough funding…”*

Another health authority added

*“The regional government distributes [money] according to its priorities. That is why the management coordinators, for example, go and fight, right? saying: ‘This is my budget and nobody touches it... because I need it for this, for that, because people with TB are going to die because pregnant women are going to die.’ So the regional government says, ‘nobody touches those budgets’ and if there is extra, they give it to the other [diseases]…”*

#### Surveillance goals and metrics: Lack of clarity guiding decision-making and planning.

According to national guidelines [19], 20% of homes at risk for triatomine infestation must be inspected annually. This target is set without considering evidence-based definitions of ‘risk area’, nor the amount of inspections conducted previously, nor the spatial coverage of inspections. Below are some excerpts from a local health authority and a VCS, depicting this lack of clarity and its impact on motivation to perform work thoroughly:

*“The [surveillance] goals remain the same; what [should] change is where the inspection is carried out. But the vector control specialist inspects in the same places because it’s easier...” (Health Authority)*

*[...] “If it is the same area, you go year after year, year after year, to a house. They tell you you have to enter [the houses], to inspect and all that. But [if you’ve been to the house before] and you know that you have never found it* [the triatomine]*, you do it a little bit faster…” (vector control specialist)*

VCSs reported that inspections take anywhere from 10 to 30 minutes, indicating significant variation in thoroughness. Additionally, a health authority noted that the national guidelines for triatomine surveillance have not been updated in decades:

*“In Arequipa we work based on our experience. The [National] Chagas standard is outdated…, it is from the year 1998 and it is in ‘the process of being updated’, but no one knows where it’s really at. For many years we have been asking for it… it has to be done by MINSA [the Peruvian Ministry of Health]”. (Health Authority)*

Regional authorities also explained that the electronic system used to report programmed activities is incomplete, so they also rely on a parallel paper-based system. This dual reporting generates further complications, as the Ministry of Health and the Ministry of Economy and Finance base budgetary decisions on the electronic system, which fails to capture the full extent of activities conducted, making it harder to secure adequate funding. Additionally, sometimes VCS visit homes for follow-up, but do not find the resident at home, there is no way to document, or receive reimbursement, for this expense. One local health authority explains in detail:

*“The State has implemented electronic systems for me to receive money and to report what I am spending. But those systems have their flaws. A lot of the information doesn’t get through. Lima tells me: ‘Hey, you are not working’, but I am working: look at my reports, look at my documents! [They respond:] ‘But I don’t have it here in the system’ because the failure starts here and it gets lost along the way… Lima enters into the [electronic] system… and says: ‘Let’s see - are you producing? No! You are not producing anything! So, what money should I give you, if you don’t need it?’ So that’s where the budget starts getting lost.” (Health Authority)*

#### Inconsistencies and limitations in the reporting system.

Our respondents also discussed barriers in reporting vector surveillance data due to a lack of standardized reporting procedures, including missing reporting codes in the Health Information System (HIS), data that HIS requests but does not report out (see first excerpt below), and the lack of a way to document visits to houses that denied entry to the VCS or were unavailable at the moment of the visit (second excerpt). Additionally, reporting practices among VCSs vary widely: some only report monthly to the vector-borne strategy authority, others only report through the HIS, and a few also report their activities in the local health plan. Additionally, one participant mentioned reporting to the local municipality:

“*The HIS problem... Do you know what the problem is? We put information in HIS, but it is not reported in the report. In other words, the electronic system takes in the information but does not report it. So, at the end of the month, I want to know how much was done, [but] nothing comes out! Why am I going to report it, if nothing comes out?*” *(Vector Control Specialist)*

Regarding the inability to report “unsuccessful” house visits (where they were unable to speak to the resident for different reasons), one VCS explains:

*“In other words, in our document, we are only asked for the number of homes inspected during the month of April, for example, 30 [houses]. But a house that did not let you go in, no, that doesn’t count, you have to go again.” (Vector control specialists)*

## Discussion

This study identified critical gaps and barriers in the processes of the vector surveillance system for Chagas disease in Arequipa. At each step, challenges emerged that hindered the system’s efficiency, including fragmented information flow, inadequate documentation and follow-up, insufficient health personnel for responses, and a lack of “reflection” in a complex and dynamic system that did not incorporate new goals or strategies. Collectively, these barriers significantly diminish the system’s potential to control the spread of triatomines effectively. Our findings are consistent with those from other regions in Latin America [20–23], where the efficacy of triatomine control programs is compromised by a lack of sustained political commitment, decentralization of disease control programs, a reduction in trained personnel and budget competition across other vector-borne disease control program [24–26].

Active surveillance for vector control in Arequipa operates on a quota system (“metas”), requiring personnel to inspect a fixed number of houses for triatomines. Quotas, and pay-for-performance schemes [27,28], are strategies imported from high income countries [29]. This situation creates a paradox: personnel are required to meet quotas to secure funding, but they need funding to cover transport and other costs necessary to meet the quotas. While the quotas come from the leadership of the vector control program, the day to day activities of the vector control specialists are under the supervision of health center managers. Vector control specialists must negotiate with their immediate managers for the resources and time they need to conduct household inspections. Managers rarely support active inspections, and often assign vector control specialists to unrelated tasks at the health center. Coordination amongst government institutions, and the tensions it generates, is a consequence of the decentralization in health [30] a reform that shifted authorities to regional, district, and municipal levels [31]. Experiences across Latin America report multiple negative impacts of decentralization, including the absence of local expertise [32,33], limited resources and organization [34], and lack of local political leadership to sustain the programs [34]. In Argentina, for example, it took 10 years following decentralization for regional programs to achieve the same coverage of Indoors Residual Spraying they had achieved before the reform [35]. In Brazil, decentralization led to unsystematic and sporadic data collection, leading to the loss of valuable epidemiological information [36]. In Minas Gerais State, Brazil, surveys conducted between 2004 and 2006 found that domestic Chagas disease vector infestation persisted due to the inefficiency of municipal programs - some of which have not conducted any control activity since decentralization [34].

Further, the quotas are simply a *number* of houses--they are agnostic in terms of *which* houses are to be inspected. Facing the additional constraint of lack of transportation funds, and limited time and support from direct superiors for active surveillance, most vector control specialists, if they conduct inspections at all, do so through convenience sampling--visiting households near the health centers, or occasionally near water treatment plants or other installations they visit for other purposes. The quota system must be de-implemented to make space for more rational and effective approaches, especially those that can make use of the wealth of spatial information, historical spray data, and other known correlates of infestation risk [37] to guide active surveillance efficiently. Neglecting such information about the factors that make certain populations more vulnerable to vectors, and providing insufficient funds for vector control specialists to reach at-risk households, contributes to reinforce inequalities in health coverage and delivery.

Unnecessary barriers and inefficiencies similarly plague the passive surveillance system. The requirement for community members to capture and bring a triatomine to a community health worker or a health center is impractical and discourages participation. Community members can be reticent, or unable, to capture the insects. They may lack time or motivation to visit a health center or may feel uncertain that their efforts will lead to an effective response. Very few community health workers are ‘active’, and those often spend more time and energy on better-funded programs. The health posts do not have a good system for receiving triatomines. Insects brought in by community members are frequently lost, mishandled, or forgotten. Although passive surveillance has been reported as a sensitive and cost-effective method for early detection of infestations [38,39], it relies on key elements, such as a close relationship between affected communities and researchers and/or surveillance staff [38], a quick response to infestation reports [40,41], as well as a sustained community motivation to participate in surveillance activities. In endemic areas where these elements are missing, community participation in passive surveillance presents similar barriers to the ones described in Arequipa [34,42]. Addressing these challenges requires revising passive surveillance protocols to simplify reporting, foster trust, and strengthen the connection between communities and health systems.

Information flow modernization is critical to an effective functioning of the surveillance and control system; being a key element to deliver health services [43], providing reliable data to support informed decisions in health [44], and improving health system response to stressors and shocks [2]. The Health Information System (HIS) in place to record triatomine surveillance and control activities in Arequipa was originally developed to manage primary healthcare data and related administrative activities [45]. It is rigid and structured and does not address the needs of the triatomine surveillance system, neither active nor passive, resulting in barriers to data recording, management, flow, and analysis. Participants report that entomological inspections and infestation data are often incomplete, geospatial information critical for planning interventions is missing, and data flows only one way, preventing analysis to adjust interventions as needed. The current data system does not allow an assessment of the impact of surveillance and control activities. Some activities are conducted but not registered, or staff might be discouraged to conduct activities that will not be visible in the system [42].

New strategies for triatomine vector surveillance and control have recently been tested in Arequipa. These show promise to build resilience for routine stressors and unexpected shocks by strengthening the system’s adaptive response and its capacity for self-regulation [46]. In a cluster randomized trial, reframing the surveillance system from a militarized, top-down system to one patterned after the immune system [13], significantly increased the system’s ability to detect and eliminate infested houses compared to a conventional approach. A new internet-based community reporting system [4], not only ensured the continuous delivery of services under an unexpected shock (the SARS-Covid pandemic) but also increased the system’s ability to detect infestations. While these strategies have proven successful in Arequipa, they clash with existing rules and policies, complicating their integration into the broader health system [2,47].

This evaluation was conducted in a specific city within a particular context; the results are not meant to be generalized across Peru, or beyond. However, the findings highlighted barriers that, although specific to the entomological surveillance system, may similarly affect other areas of the Peruvian health system [48], as well as vector surveillance systems elsewhere [20,40]. In addition, we could not interview health authorities at the national level, which leaves a gap in understanding the higher-level policy decisions that shape the implementation of surveillance systems. We also faced significant challenges in interviewing community members who had reported infestations, as many were reluctant to participate, while others were unavailable. This further limits the scope of our findings, particularly regarding community-level perspectives Furthermore, the limited number of social and community health workers available for interviews (due to their scarcity) constrained our ability to explore their perspectives and experiences comprehensively.

Vector-borne diseases are dynamic systems– changes in society, the environment, and the vector or pathogens themselves, require new strategies and interventions. When a strategy works it can shift the epidemiologic scenario –requiring a constant cycle of innovation and assessment [49]. Designing such a system requires participatory construction or “co-design”, from affected communities to health authorities [2]. At the policy level, efforts towards designing interventions together with other government sectors are fundamental [46,50]. Vulnerability to Chagas disease vectors is ultimately generated by social forces [51–54]. Policies to address these factors are essential to alleviate the disease burden in affected populations and in the health systems in general.

## Supporting information

S1 File Interview guide used for semi-structured interviews with household members where kissing bugs and bed bugs are reported. (PDF)

S2 File Consolidated criteria for reporting qualitative research (COREQ) checklist used for the paper. (PDF)

S3 Striking image caption. (DOCX)

S4 striking_image. (TIF)
