# Supplementary material for: Barriers to surveillance and control of re-emergence of the Chagas disease vector Triatoma infestans in Arequipa, Peru
Source: PLoS Negl Trop Dis. 2025 Aug 7;19(8):e0013373. doi: 10.1371/journal.pntd.0013373 (PMC12331067; doi:10.1371/journal.pntd.0013373)
Supplement: S1 File — (PDF) [file pntd.0013373.s001.pdf]

Discussion Guideline for semi- structured interviews with household members where kissing bugs and bed bugs are reported

The instructions/recommendations for the discussion facilitator are in italics

Recently a member of this house identified the presence of kissing bugs and/or bed bugs in your home and made a report, so I would like to spend some time talking to you about what you have done since you identified the insects. There are no right or wrong answers to these questions, I am just interested in your opinion and hearing some details about it.

Detection and identification

1. Besides you, what other people live in this household?
2. Did you identify the presence of kissing bugs or (bed bugs)? or was it other household member?
    a. IF OTHER: Who?
        i. Is this person at home now?
        ii. What time could I return to meet him/her?

(TRY TO TALK TO THE PERSON WHO IDENTIFIED THE INSECT)
if the interviewee identified them:

3. Did you observe the insect live or its tracks (*eggs, skins or parts*)?
4. Where in your house did you observe the kissing bug, bed bug or its traces? (*e.g., "room, kitchen, yard, etc.")*
5. Do you remember approximately how long ago you saw it?
    i. *If it is difficult for the interviewee to say a date, help him or her to remember by trying to associate important moments or events that have happened recently. Note the dates of these events but not the details of them.*
6. What were the insect or the traces that you saw like?
    i. *Show illustrations or plaques of the insect and its traces*
7. What did you do when you saw the insect(s)?
    i. *Capture them*
    ii. *You took a picture*
    iii. *You killed it*
    iv. *You used insecticides*

IF YOU CAN NOT TALK TO THE PERSON WHO IDENTIFIED THE INSECT:
if the insect was identified by a different person :

8. Do you know if [*Person who identified them*] observed the live insect or its traces (*eggs, skins or parts*)?
9. Where in the house did [*Person who identified them*] tell you he/she observe the kissing bug, bed bug or its traces? (*e.g., "room, kitchen, yard, etc.")*
10. Do you remember approximately how long ago the [*Person who identified them*] mentioned you that he/she has observed these insects or its traces?

   i. *If it is difficult for the the interviewee to say a date, help him or her to remember by trying to associate important moments or events that have happened recently. Note the dates of these events but not the details of them.*

11. Did [*Person who identified them*] tell or show you the insect or the traces he/she observed? Do you remember what it looked like?
    i. *Show illustrations or plaques of the insects and its traces if the interviewee has seen the insect or its traces.*
12. Do you know what [*Person who identified them]* did after seeing these insects or its traces?
    i. *Captured them*
    ii. *He/she took a picture*
    iii. *He/she killed it*
    iv. *He/she used insecticides*

13. Who made the report about the presence of the insect in your home?
    i. *If they did not make a report, check the reason (e.g., due to unawareness, lack of time…)*

Continue only if the report was made

14. To whom did you, or [*Person who reported*], report?
    i. *Your community health worker?¿Using photos or verbally?*
    ii. *At your health facility?*
    iii. *Did you tell an inspector?*

15. Do you remember approximately how long it took from the time you saw the insect or its traces until you made the report?

16. How long did it take from the time you made the report to the time a vector control specialist came to inspect your home?
    i. *Ask questions to help him/her to remember*
    ii. What do you think of this time?
    iii. What do you think of the answer you got?

17. When the vector control specialist came to your home to inspect it, could he/she find more insects or its traces?

Discussion Guidelines for semi-structured interviews with community health workers

The instructions/recommendations for the discussion facilitator are in italics

The objective of this interview is to learn about the experiences, perceptions, and opinions of the community health workers (CHW) about their work and the detection of kissing bugs or bed bugs in the houses of Arequipa. There are no right or wrong answers to these questions, you have experiences that we want to hear. You are the ones who have done this activity--not us--and therefore we want to hear from you!

Review the consent form and obtain consent before you begin.

1. How long have you been working as a Community Health Worker?

2. What inspired or motivated you to become a Community Health Worker?

3. During your work as a Community Health Worker, what challenges or barriers have you encountered in carrying out your responsibilities?

4. Have you ever been involved in activities related to Chagas disease or triatomine bugs? If so, could you describe those experiences?

5. Have you receive training on Chagas disease and triatomine bugas? If so, How frequently it was?

6. Have you ever received a report from a community member about the presence of triatomine bugs in their home?
    - How long ago did you receive this report?
    - What actions did you take after receiving the report?
    - What happened after you reported the presence of triatomine bugs (sometimes referred to as "chirimachas")?

7. In your view, what could be done to make your work as a Community Health Worker easier?
    - More training? about what?
    - More materials? about what?
    - More technical support? what kind?
