# Supplementary material for: Barriers to surveillance and control of re-emergence of the Chagas disease vector Triatoma infestans in Arequipa, Peru
Source: PLoS Negl Trop Dis. 2025 Aug 7;19(8):e0013373. doi: 10.1371/journal.pntd.0013373 (PMC12331067; doi:10.1371/journal.pntd.0013373)
Supplement: S2 File — (PDF) [file pntd.0013373.s002.pdf]

Supplementary Material S2

A Consolidated criteria for reporting qualitative studies (COREQ): 32-item checklist

Developed from:
Tong A, Sainsbury P, Craig J. Dr *International Journal for Quality in Health Care*. 2007. 19 (6): pp. 349 – 357

No. Item	Guide questions / description	Reported on Page #
**Domain A: Research team and reflexivity**		
Personal Characteristics		
1. Interviewer/facilitator	Which author/s conducted the interview or focus group?	page 5; rows: 121-123<br><br>*"We (LDT, VPS, RG, CCP) conducted in-depth interviews using process maps and flow charts illustrating the passive and active surveillance systems in Arequipa."*
2. Credentials	What were the researcher's credentials? E.g., PhD, MD	Interviews were conducted by a female social scientist with ample experience in qualitative research with a PhD in Public Health (VPS), a female biologist with MSc in Life Sciences and MSc in Epidemiology (LDT), a female biologist with PhD in Life Sciences (RG) and a male biologist responsible for coordinating field work (CCP).
3. Occupation	What was the researcher's occupation at the time of the study?	At the time of the study, all members of the research team were working as researchers, except for Fernando Málaga, who served as the Vector-Borne Diseases Strategy Coordinator at the Ministry of Health in Arequipa.
4. Gender	Was the researcher male or female?	Interviews were conducted by 3 females (VPZ, LDT & RG and a male (CCP).
5. Experience and training	What training experience did the researcher have?	Interviews were conducted by a female social scientist with ample experience in qualitative research with a PhD in Public Health (VPS), a female biologist with MSc in Life Sciences and MSc in Epidemiology (LDT), a female biologist with PhD in Life Sciences (RG) and a male biologist responsible for coordinating field work (CCP).
Relationship with participants		
6. Relationship established	Was a relationship established	Page 5; rows: 113-115

	prior to study commencement?	*"Recruited personnel from the Ministry of Health and community health workers have been working with the research team for many years, but there was no prior relationship with community members."*
7. Participant knowledge of the interviewer	What did the participants know about the researcher? e.g., personal goals, reasons for doing the research	Prior to interviews, researchers introduced themselves to participants, providing information about their affiliations, roles, and overall research objectives.
8. Interviewer characteristics	What characteristics were reported about the interviewer/facilitator? e.g., Biases, assumptions, reasons and interests in the research topic	Page 5; rows: 117-118<br><br>*"Our objective was explicitly communicated repeatedly before and during the interviews: to establish a collective understanding of systemic barriers for triatomine vector surveillance and control."*
**Domain B: study design**		
Theoretical framework		
9. Methodological orientation and Theory	What methodological orientation was stated to underpin the study? e.g., grounded theory, discourse analysis, ethnography, phenomenology, content analysis	page 5; rows 121-125<br><br>*"We (LDT, VPS, RG, CCP) conducted in-depth interviews using process maps and flow charts illustrating the passive and active surveillance systems in Arequipa (Figure 1). These visual aids facilitated discussions with interviewees, encouraging them to specify the challenges at each step of the vector surveillance process"*
Participant selection		
10. Sampling	How were participants selected?	Page 5-row: 109-110<br><br>*"We used purposive sampling to obtain a range of perspectives regarding different components of the passive and active surveillance system. Interviews were conducted until saturation was reached."*
11. Method of approach	How were participants approached?	Page 5. rows 120-121<br>*"Interviews were conducted in a private space of individuals' workplace and, in the case of*

		community health workers and community members, in their homes to ensure privacy."
12. Sample size	How many participants were in the study?	Page 6. Table 1<br><br>32 participants were recruited.
13. Non-participation	How many people refused to participate or dropped out? Reasons?	Page 6 rows 157-158<br><br>"*None of the individuals invited to participate refused to take part in the study*"
Setting		
14. Setting of data collection	Where was the data collected?	Page 5. rows 120-121<br><br>*"Interviews were conducted in a private space of individuals' workplace and, in the case of community health workers and community members, in their homes to ensure privacy."*
15. Presence of non-participants	Was anyone else present besides the participants and researchers?	Page 6; rows: 132-134<br><br>Non-participats were not present during the interviews.<br><br>*"Interviews were conducted in a private space of individuals' workplace and, in the case of community health workers and community members, in their homes to ensure privacy"*
16. Sample description	What are the important characteristics of the sample?	Page 6,Table 1
Data collection		
17. Interview guide	Were questions, prompts, guides provided by the authors? Was it pilot tested?	Page 6; rows: 129-1133<br><br>*"Some interviewees, especially community members, community health workers, and social workers (who oversee the community health workers), had limited awareness of the surveillance system processes. We, therefore, developed a structured interview guide tailored to these participants, focusing on their experiences and challenges within the surveillance system (Supplementary material 1)."*

18. Repeat interviews	Were repeat interviews carried out? If so, how many?	All interviews were applied a single time.
19. Audio/visual recording	Did the research use audio or visual recording to collect the data?	Page 6; rows: 141-142<br>*"We audiotaped and transcribed all interviews and the focus group. Only one respondent requested not to be audiotaped, so we took detailed notes instead."*
20. Field notes	Were field notes made during and/or after the interview or focus group?	Page 6; rows: 142-144<br>*"As the interviews progressed, either the interviewer or the respondent filled out blank process maps used to guide discussions on barriers in passive and active surveillance."*
21. Duration	What was the duration of the interviews or focus group?	On average, interviews with health authorities, VCS, and social workers last about one hour, while interviews with community health agents and community members are briefer, lasting between 3 and 7 minutes. The focus group session lasted about 2 hours.
22. Data saturation	Was data saturation discussed?	Page 5; rows: 110<br>*"Interviews were conducted until saturation was reached."*
23. Transcripts returned	Were transcripts returned to participants for comment and/or correction?	Page 6; rows: 135-137<br>*"Although interview transcripts were not returned to participants for review, we held a focus group with vector control specialists to present findings and discuss potential solutions tailored to their unique realities."*
**Domain C: analysis and findings**		
Data analysis		
24. Number of data coders	How many data coders coded the data?	Page 6; row 147-148<br>*"A team of four individuals double-coded each transcript, refining the codebook iteratively to ensure consistency and accuracy in identifying key themes."*

25. Description of the coding tree	Did authors provide a description of the coding tree?	Page 6; rows: 144-147<br><br>*"Based on the initial reading of transcripts, we developed a codebook using both deductive codes (steps of the process maps) and inductive codes (emerging themes).This iterative process resulted in a final code tree, which included categories and subcategories illustrated on flow charts."*
26. Derivation of themes	Were themes identified in advance or derived from the data?	Page 6; row 147-148<br><br>*"A team of four individuals double-coded each transcript, refining the codebook iteratively to ensure consistency and accuracy in identifying key themes."*
27. Software	What software, if applicable, was used to manage the data?	Page 6; rows: 148-149<br><br>*"Interviews and focus group transcripts were managed, coded, and analyzed using Dedoose (version 9.0.90)."*
28. Participant checking	Did participants provide feedback on the findings?	Page 6; rows: 135-137<br><br>*"Although interview transcripts were not returned to participants for review, we (LDT, VPS) held a focus group with vector control specialists to present findings and discuss potential solutions tailored to their unique realities."*
Reporting		
29. Quotations presented	Were participant quotations presented to illustrate the themes/findings? Was each quotation identified? e.g., participant number	Manuscript - Results (page 7 to 16)<br>Yes, representative quotations from participants are included in the Results section to illustrate themes.
30. Consistency of data and findings	Was there consistency between the data presented and the findings?	Yes, there was consistency between the data presented and the findings.<br>Results (page 7 to 16) and Discussion (page 17 to 20)
31. Clarity of major themes	Were major themes clearly presented in the findings?	Yes, the major themes were clearly present in the findings.<br>Results (page 7 to 16) and Discussion (page 17 to 20)

32. Clarity of minor themes	Is there a description of diverse cases or discussion of minor themes?	Yes, minor themes were also discussed in the findings.<br>Results (page 7 to 16) and Discussion (page 17 to 20)
