# Supplementary material for: Barriers to surveillance and control of re-emergence of the Chagas disease vector Triatoma infestans in Arequipa, Peru
Source: PLoS Negl Trop Dis. 2025 Aug 7;19(8):e0013373. doi: 10.1371/journal.pntd.0013373 (PMC12331067; doi:10.1371/journal.pntd.0013373)
Supplement: S3 — (DOCX) [file pntd.0013373.s003.docx]

STRIKING IMAGE CAPTION:

Engorged *Triatoma infestans* nymph.

Credits: Photo: Raquel Gonçalves; Image edited by: Gustavo Suárez.
