# Supplementary figures and images for: Barriers to surveillance and control of re-emergence of the Chagas disease vector Triatoma infestans in Arequipa, Peru
Source: PLoS Negl Trop Dis. 2025 Aug 7;19(8):e0013373. doi: 10.1371/journal.pntd.0013373 (PMC12331067; doi:10.1371/journal.pntd.0013373)

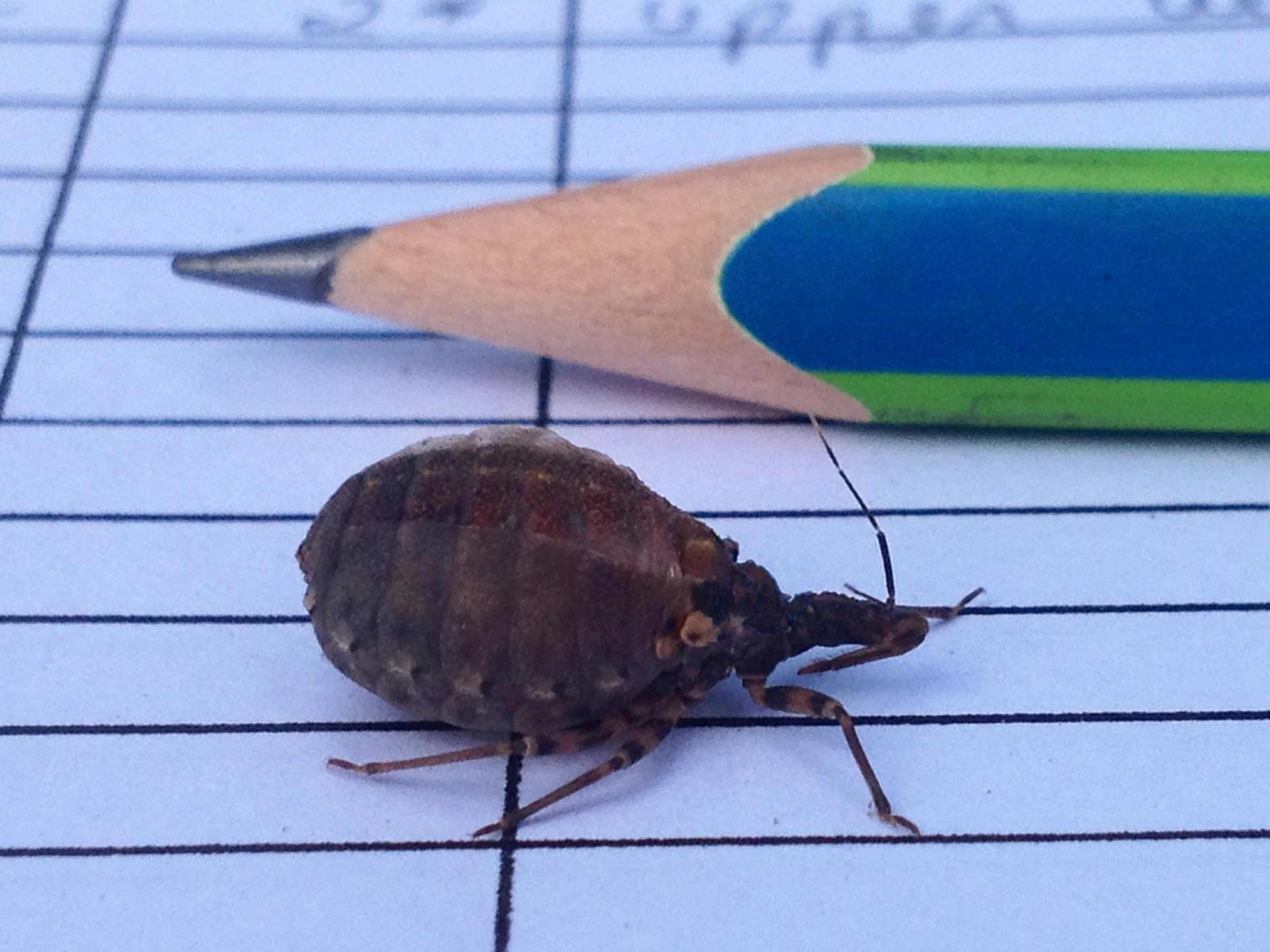

Supplement: S4 — (TIF) [file pntd.0013373.s004.tif]
